# Characterization of Marine Aerosol for Assessment of Human Exposure to Brevetoxins

**DOI:** 10.1289/ehp.7496

**Published:** 2005-02-09

**Authors:** Yung Sung Cheng, Yue Zhou, Clinton M. Irvin, Richard H. Pierce, Jerome Naar, Lorraine C. Backer, Lora E. Fleming, Barbara Kirkpatrick, Dan G. Baden

**Affiliations:** ^1^Lovelace Respiratory Research Institute, Albuquerque, New Mexico, USA;; ^2^Mote Marine Laboratory, Sarasota, Florida, USA;; ^3^Center for Marine Science, University of North Carolina at Wilmington, Wilmington, North Carolina, USA;; ^4^Centers for Disease Control and Prevention, Atlanta, Georgia, USA;; ^5^National Institute of Environmental Health Sciences Marine and Freshwater Biomedical Science Center, University of Miami, Miami, Florida, USA

**Keywords:** brevetoxin, exposure assessment, *Karenia brevis*, marine aerosol, particle size distribution, personal exposure, red tide

## Abstract

Red tides in the Gulf of Mexico are commonly formed by the fish-killing dinoflagellate *Karenia brevis*, which produces nine potent polyether brevetoxins (PbTxs). Brevetoxins can be transferred from water to air in wind-powered white-capped waves. Inhalation exposure to marine aerosol containing brevetoxins causes respiratory symptoms. We describe detailed characterization of aerosols during an epidemiologic study of occupational exposure to Florida red tide aerosol in terms of its concentration, toxin profile, and particle size distribution. This information is essential in understanding its source, assessing exposure to people, and estimating dose of inhaled aerosols. Environmental sampling confirmed the presence of brevetoxins in water and air during a red tide exposure period (September 2001) and lack of significant toxin levels in the water and air during an unexposed period May 2002). Water samples collected during a red tide bloom in 2001 showed moderate-to-high concentrations of *K. brevis* cells and PbTxs. The daily mean PbTx concentration in water samples ranged from 8 to 28 μg/L from 7 to 11 September 2001; the daily mean PbTx concentration in air samples ranged from 1.3 to 27 ng/m^3^. The daily aerosol concentration on the beach can be related to PbTx concentration in water, wind speed, and wind direction. Personal samples confirmed human exposure to red tide aerosols. The particle size distribution showed a mean aerodynamic diameter in the size range of 6–12 μm, with deposits mainly in the upper airways. The deposition pattern correlated with the observed increase of upper airway symptoms in healthy lifeguards during the exposure periods.

Red tides in the Gulf of Mexico are commonly formed by the fish-killing dinoflagellate *Karenia brevis* (formerly known as *Gymnodinium breve*). The organism produces as many as nine potent polyether brevetoxins called PbTxs and designated PbTx-1, PbTx-2, etc., that result in the death of a massive number of fish (Forrester et al. 1977), mammals (Bossart et al. 1998), and other marine species during red tide blooms. PbTxs can also concentrate in the tissues of shellfish that feed on dinoflagellates. People who eat these shell-fish may suffer from neurotoxic shellfish poisoning, a food poisoning syndrome that can cause severe gastrointestinal and neurologic symptoms.

Brevetoxins can also be transferred from water to air in the wind-powered white-capped waves during red tide episodes (Pierce et al. 1989a). Inhalation exposure to marine aerosol containing brevetoxins causes respiratory symptoms, including involuntary coughing and sneezing, watery eyes, rhinorrhea, a burning sensation in the throat and nose, and difficulty breathing (Backer et al. 2003; Cheng et al. 2005; Pierce et al. 2003). Red tides off Florida coastal waters are almost annual events. Historically, anecdotal reports and limited references to human symptoms in the literature (Music et al. 1973; Woodcock 1948) have consistently cited acute respiratory and eye irritations as typical responses from exposure to aerosolized PbTx. In addition, experimental work (Abraham et al. 2004; Asai et al. 1982; Baden et al. 1982) demonstrated that inhaled PbTx could cause bronchoconstriction and a smooth respiratory muscle response that could result in an asthma attack in susceptible individuals.

During two red tide events in Florida in 1999, PbTx levels in air and seawater were measured and personal interviews and pulmonary function tests were conducted on people before and after they visited Florida beaches (Backer et al. 2003). During moderate- and high-exposure periods, 36 and 80 ng/m^3^, respectively, of PbTx were detected in the air. Lower respiratory tract symptoms (e.g., tightness of chest, wheezing, shortness of breath) were reported by 8% of the people who had no or low exposure, 11% with moderate exposure, and 28% with high exposure; upper respiratory symptoms (eye and throat irritation, nasal congestion, cough) were also increased in the moderate- and high-exposure groups. Nasal-pharyngeal swabs were taken from people who experienced moderate or high exposure, and we found a mild inflammatory response in > 33% of these participants. This initial study indicated that brief recreational exposure to red tide aerosol by beach goers could be related to increased respiratory symptoms. We also found in the fall of 2000 in a red tide event near Corpus Christi, Texas, that low air concentrations of brevetoxins in the range of 3–4 ng/m^3^ could initiate symptoms in the upper respiratory tract, including cough, irritation in the throat, and itchy eyes (Cheng et al. 2005).

Red tide aerosols are marine aerosols containing aerosolized *K. brevis* fragments and associated bacteria (Pierce et al. 1989a). Environmental aerosol samples collected during red tides in Florida and North Carolina in 1987 (Pierce et al. 1989b) showed high concentrations of three PbTxs (PbTx-2, PbTx-3, and PbTx-5). Generation of red tide aerosol was also studied in the laboratory using cultures of *K. brevis* (Pierce et al. 1989a, 1989b). In general, similar PbTxs profiles were observed in the laboratory and the field.

Characterization of the red tide aerosols in terms of its concentration, chemical composition, toxin profile, and particle size distribution is essential in understanding the source, assessing human exposure, and estimating dose of inhaled aerosol. With additional information on local weather conditions and water samples in the bloom, one can also establish a relationship between the aerosol concentration and environmental conditions. We describe in this article a detailed characterization of aerosols and environmental conditions during an epidemiologic study of occupational exposure to Florida red tide aerosol.

## Materials and Methods

### Field sampling study.

The occupational exposure study was conducted in Sarasota, Florida. The experimental design had two 5-day study periods including an exposure period between 7 and 11 September 2001 during a red tide episode and a nonexposure period between 3 and 7 May 2002. The detailed study design for the epidemiologic study is described in this mini-monograph by Backer et al. (2005). Air samplers were set up along Siesta Beach and Lido Beach (Sarasota, Florida) to collect marine aerosols. High-volume air samplers (model G2000H; Andersen Instruments, Smyrna, GA) were placed near the water to collect large quantities of material for analysis of PbTx. Some samplers collected airborne particles in one filter substrate for total aerosol concentration, whereas other samplers housed a five-stage, high-volume cascade impactor (model SA235, Andersen Instruments) for total concentration as well as particle size distribution. Glass-fiber filters (20 cm × 25 cm) were used as the collection substrates (model EPM2000; Whatman International Ltd., Maidstone, UK) for the single-stage filter sampler. Cellulous filters (15 cm × 14 cm; filter paper 41, Whatman) were used for the five-stage cascade impactor. There were one impactor sampler and three filter samplers at Siesta and Lido beaches. The sampling time usually started between 0800 and 0830 hr and ended between 1530 and 1630 hr. We also ran one impactor sampler at night for a total of approximately 15 hr at Siesta Beach during the exposed period of 7–10 September 2001. The samples collected represented the time-integrated ambient concentration and particle size in the sampling area.

Seawater samples were collected in 1-L glass bottles 3 times each day (0830, 1200, and 1600 hr) from the surf zone adjacent to each air sampler location to provide an indication of changes in cell counts and toxin composition throughout each day. A 20-mL subsample was collected from each bottle and fixed with Utermohl’s solution for microscopic identification and enumeration of *K. brevis* cells. The remaining water sample was processed for brevetoxin analysis by liquid chromatography–electrospray ionization mass spectrometry (LC-MS) and for verification by enzyme-linked immunosorbent assay (ELISA).

### Personal exposure.

Personal exposure was measured by the occupational study participants who wore a personal sampler (IOM inhalable dust sampler; SKC, Inc., Eighty Four, PA) connected to a battery-operated pump (Hi Flow Sampler; Gillian Instrument, Wayne, NJ). The sampler was placed at the lapel near the breathing zone. A 25-mm glass fiber filter (type A/E; Pall Life Science, Ann Arbor, MI) was used as the collection substrate. The sampling flow rate was 2-L/min controlled by a rotameter in the sampling pump.

### Monitoring of weather conditions.

A portable, solar-powered weather station was deployed at Siesta Beach near the impactor sampler to provide wind speed and direction, temperature, and relative humidity (Complete Weather Station; Davis Instruments, Hayward, CA). It continued to monitor weather conditions during the sampling period, and the data were downloaded daily. The wind direction measured at Siesta Beach included 16 quadrants. We assigned arbitrary values for onshore wind (1 for W, WSW, SW, SSW, and S), offshore wind (0 for ESE, E, ENE, NE, NNE, and N), and partially onshore wind (0.5 for SSE and WNW, 0.3 for NW, 0.1 for NNW and SE). The daily average of the direction was then the mean value averaged over the sampling period; the standard deviation was a measure of the variability in wind direction measurement. The mean wind direction can also be viewed as the fraction of time the wind direction was onshore.

### Analysis for air samples.

After collection, the high-volume impactor substrates were stored at −20°C. Filters were removed from the cold storage and equilibrated to room temperature before extraction. First, substrates were cut into several sections. About 3.0 cm^2^ was used for ELISA analysis for total concentration of brevetoxins and related compounds. About 90 cm^2^ of samples were extracted and analyzed by LC-MS.

The extraction and LC-MS techniques have been described in detail (Cheng et al. 2005). Briefly, the section of filter for LC-MS-MS analysis was extracted first by folding over and rolling it into a 15-mL polypropylene tube. Ten milliliters of acetone were added to the tube and the sample was vortexed for 20 sec, sonicated for 2 min, and then placed on a circular rotator (Roto-Torque, low speed #10; Cole-Parmer Instruments, Vernon Hills, IL) for 20 min. The 10-mL extract was then evaporated under a gentle stream of nitrogen to approximately 100 μL, vortexed for 5 sec to rehomogenize the extract, and recombined with a 50:50 methanol:purified water to the final analysis volume (typically 200 μL). The samples were then analyzed for brevetoxins by an LC-MS technique using a high-performance liquid chromatograph (SIL-DAD vp; Shimadzu Co., Kyoto, Japan) coupled with the API 365 MS/MS (Applied Biosystems Inc., Foster City, CA). We ran the instrument in multiple-response monitor mode and used the parent/daughter ion pairs of 867.5/849.5 (PbTx-1), 895.5/877.5 (PbTx-2), 897.5/725.4 (PbTx-3), 911.6/893.7 (PbTx-6), 899.6/863.9 (PbTx-9), and 657.4/273.2 (brevenal) to identify and quantify the PbTx components. The limit of quantitation for the analysis of the impactor sample was 0.01 ng/m^3^.

Filter samples from personal samples and small sections of each impactor substrate were analyzed by a competitive ELISA (Naar et al. 2002) based on the specific activity of the goat anti-PbTx antibody (Trainer and Baden 1991). The analysis provided a total amount of PbTxs but not the individual PbTxs. The limit of quantitation of the brevetoxins using the ELISA assay was 0.6 ng/sample. For the personal samples, this corresponded to about 1 ng/m^3^ for a sampling period of 300 min.

### Analysis of water samples.

We extracted brevetoxins from the water samples by passing the seawater through a C_18_ solid-phase extraction disk under vacuum (Ansys Technologies, Inc., Lake Forest, CA) according to the procedure of Pierce et al. (2003). The C_18_ disks were then rinsed with reverse osmosis water to remove any remaining salts and eluted with methanol for LC-MS and ELISA analyses. We verified the extraction efficiency by recovery of standard toxins added to seawater samples and processed as described above.

Brevetoxin analyses of water samples were performed at the Mote Marine Laboratory by LC-MS using a ThermoFinnigan AqA LC-MS. The LC consisted of a SpectraSystems: LC pump P4000, autosampler AS3000, and degasser SCM1000 (Thermo Electron Co., Waltham, MA). Mass spectral detection was obtained using an AqA single quad system scanned from 204 to 1,216 AMU with AqA Max 40 V, and a scan rate of 1.1 scans/sec. All analysis was conducted using electrospray with the probe at 3 kV and 250°C. The column was a Phenomenex Security Guard C_18_ guard column with a Phenomenex Luna C_18_ 5Fm 250 × 2 mm analytical column (Phenomenex USA, Torrance, CA). The solvent gradient was composed of acidified (0.3% acetic acid) acetonitrile (ACN)/H_2_O with initial 50:50 ACN/H_2_O to 95:5 ACN/H_2_O over 40 min.

### Dose estimation of inhaled PbTxs.

The deposition pattern of the inhaled red tide aerosol in different regions of the respiratory tract can be estimated using the International Commission on Radiological Protection (ICRP) 66 lung model (ICRP 1994). The human respiratory tract is divided into three anatomical regions. The extrathoracic (ET) airway including the naso-oro–pharyngo–laryngeal region is the entry to the respiratory tract and the first defense against hazardous inhaled material. The tracheobronchial (TB) tree region includes the trachea and 16 generations of branching airways. Gas exchange takes place in the pulmonary region. Particles deposit in the lung by inertial impaction, sedimentation, diffusion, and electrostatic mechanisms.

Assuming a breathing rate of 25 L/min for light exercise of an adult male and measured particle size distribution, we calculated the deposition fractions of inhaled particles in the three regions of the human respiratory tract using LUDEP software (NRPB, Oxon, UK), which is based on the ICRP lung model (ICRP 1994).

### Statistical analysis.

A paired *t*-test was used to determine whether differences of mass median aerodynamic diameter (MMAD) between the LC-MS and ELISA analysis were significant. A *p*-value < 0.05 was considered significant.

## Results

### Environmental conditions.

Table 1 summarizes the environmental data obtained for the two sampling periods during the 2001–2002 Occupational Exposure Study in Siesta Beach, Florida. The data showed that during the two sampling periods, the temperature and relative humidity were stable. In addition to temperature and relative humidity, the wind speed [in miles per hour (mph)] and wind direction were measured.

### PbTx concentration in air and water.

Environmental sampling confirmed the presence of *K. brevis* in water and brevetoxins in the water and air during the red tide exposure period (September 2001), and the lack of significant toxin levels in the water and air during the unexposed period (May 2002) (Table 2). Surf water samples from the non-exposed May 2002 period contained low concentrations of brevetoxins, ranging from below the limit of quantitation (0.05 μg/L) to 0.3 μg/L for samples collected from 3 to 7 May 2002 in both Lido and Siesta beaches. Air concentrations were expressed as nanograms per cubic meter of PbTxs (LC-MS technique) and were the mean value of three filter and one impactor samples collected at Lido and Siesta beaches. During the nonexposure period, air samplers recovered trace amounts of brevetoxins from the air ranging below the detection limit of quantitation (< 0.01 ng/m^3^) to 1 ng/m^3^.

Water samples collected during a red tide bloom showed moderate-to-high concentrations of *K. brevis* cells and PbTxs. The daily mean total PbTx concentration and standard deviation in water samples ranged from 8 to 28 μg/L from 7 to 11 September 2001. The mean concentrations were between 18 and 28 μg/L for the first 2 days. The aerosol concentrations were between 1.9 and 12 ng/m^3^ at the Siesta Beach and 1.3 and 27 ng/m^3^ at the Lido Beach. Higher concentrations (> 8.6 ng/m^3^) were reported between 7 and 9 September 2001, in general corresponding to higher PbTx concentrations in water and onshore wind direction. On 10 and 11 September 2001, the wind directions were mostly offshore, resulting in lower air concentrations (< 6 ng/m^3^). We also analyzed evening samples collected on impactors at night between 7 and 10 September 2001. The air concentrations of PbTx were very low: from below limit of quantitation (0.01 ng/m^3^) to 0.32 ng/m^3^. Weather conditions were not monitored, nor were water samples taken at night.

**Figure 1:**
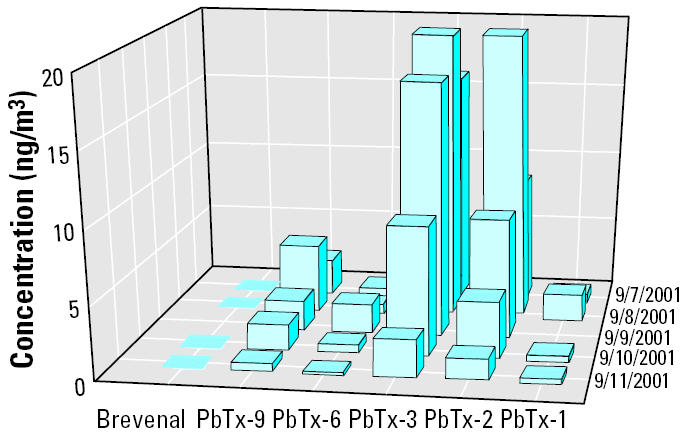
PbTx profile in the impactor samples collected at Lido Beach in September 2001.

The brevetoxin profiles in aerosol samples taken during the month of September 2001 at Lido Beach are shown in Figure 1. These data show that PbTx-2 and PbTx-3 were the major brevetoxin species present. Much lower concentrations of PbTx-1, PbTx-6 and PbTx-9 were also observed. Trace amounts of brevenal, a natural brevetoxin antagonist, were also detected in water and air samples. The brevenal was recently isolated from *K. brevis* culture by our colleagues at the University of North Carolina at Wilmington (Bourdelais et al. 2004) and was beneficial in preventing airway responses in the laboratory animal study (Abraham et al. 2004).

### Particle size distribution.

**Figure 2:**
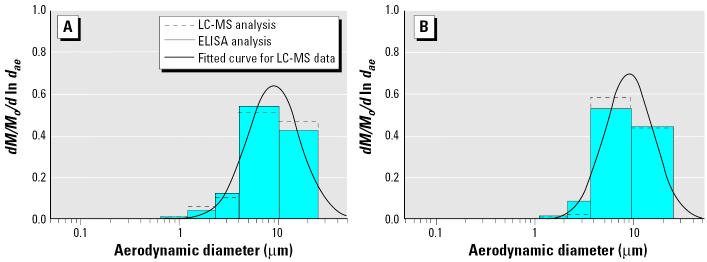
Particle size distribution of red tide aerosol based on LC-MS and ELISA analysis of impactor sample for (*A*) Siesta Beach on 8 September 2001 and (*B*) Lido Beach on 8 September 2001. Abbreviations: *d_ae_*, aerodynamic diameter; *M*, PbTx mass collected on each stage; *M_o_*, total mass of the impactor sample.

Particle size distributions of red tide aerosol were estimated from impactor samples, which were analyzed by both the LC-MS and ELISA techniques. Figure 2A and B shows normalized size distributions on samples obtained 8 September 2001 at Siesta and Lido beaches, respectively. Our data showed that the particle size distributions as analyzed by both ELISA and LC-MS analysis were similar. The size distribution showed a range of particles from 0.5 to 20 μm with a single-size mode approximately 6–12 μm. As shown in Figure 2A and B, the particle size can be described with a lognormal size distribution:


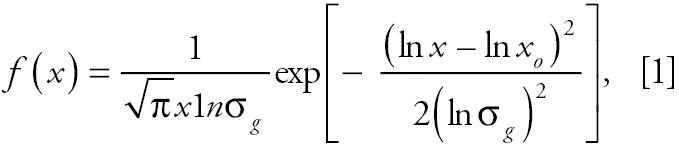


where *x*_o_ is the MMAD and σ_g_ is the geometric standard deviation (GSD). The best-fit curve using SigmaPlot software (Version 8.0; SPSS, Chicago, IL), as shown in Figure 2A and B, has an MMAD of 9.1 and 8.9 μm and a GSD of 1.85 and 1.76 for Siesta and Lido beaches, respectively, based on LC-MS analysis. The MMADs and GSDs of the fitted log-normal distribution of red tide aerosols are listed in Table 3. The MMAD obtained from LC-MS and ELISA analyses was not significantly different for both Siesta and Lido beaches. This particle size information was then used to estimate the dose of inhaled red tide aerosol in the human volunteers (see below). The concentration of PbTx was too low to estimate particle size distribution for samples obtained during the nonexposed period of May 2002.

### Dose calculation.

The fractional deposition of inhaled red tide aerosol in the human respiratory tract was estimated based on the size measurements as listed in Table 3. A total deposition fraction of 76–90% was calculated, with the majority of aerosol deposited in the ET region or upper airway (75–84%), and small but not insignificant deposition (2–6%) in the lower airways (TB and pulmonary region). The inhaled red tide aerosol has high deposition efficiency in the respiratory tract; thus, the pattern of deposition would help to explain the observed respiratory symptoms. The dose rate of the deposited brevetoxins can be calculated from Table 3 for a breathing rate of 25 L/min and for a unit air concentration of 1 ng/m^3^ of PbTx, as shown in Table 4.

### Correlation of PbTx concentration with environmental factors.

To understand the contribution of various parameters that may influence the air concentration of red tide aerosol, we analyzed further the data obtained at Siesta Beach where the weather information was measured during both the 2001 and 2002 sampling period. The total brevetoxin concentration obtained from the impactor sample was modeled as a function of water concentration, wind speed, and wind direction monitored near the impactor sampling site. The following empirical relationship was developed:


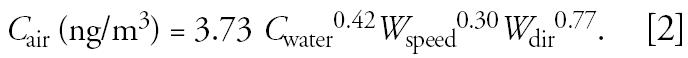


**Figure 3:**
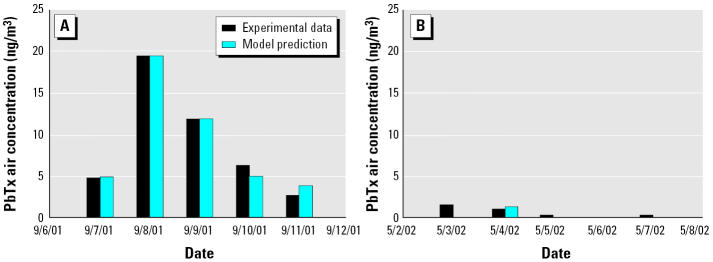
Comparison of PbTx concentrations of red tide aerosol observed in Siesta Beach and model calculations (Equation 2) for (*A*) September 2001 and (*B*) May 2002 sampling periods.

This empirical model indicates the relative importance of water concentration, wind speed, and wind direction on the red tide aerosol concentration. The four constants in this equation were obtained by a nonlinear regression procedure of SigmaPlot software. An *R*^2^ value of 0.99 indicates a very good fit between the model and the experimental data (Figure 3).

### Personal samples.

**Figure 4:**
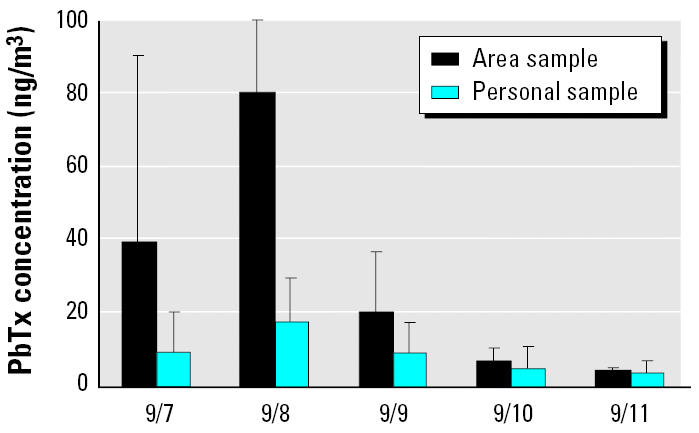
PbTx concentration (mean ± SD) as determined from area and personal samples in September 2001.

During the occupational study conducted in 2001 and 2002, personal samples were worn by seven or eight researchers and by lifeguards working on the Siesta and Lido beaches for about 8 hr a day. All 39 personal samples taken during the sampling period of 3–7 May 2002 showed no detectable amount of PbTx. During the exposure period from 7 to 11 September 2001, a total of 39 personal samples (mean sampling time, 471 min) from both the Siesta and Lido beaches were analyzed. The exposure concentration of PbTx is shown in Figure 4. Our results indicated a large variability of personal samples within each sampling day. Comparison of PbTx concentration obtained from area samples taken with high-volume samplers showed that the personal samples indicated a lower exposure concentration (on the average about 50%) from those obtained from the area sample. However, the daily means of the exposure concentrations from the personal samples followed the concentrations obtained from the area samples. This may be attributable to different sampling techniques with much lower flow rate for personal samplers and the possibility that the subjects spent only part of the time in the exposure area. This was the case for both the researchers and lifeguards, who were in and out of the beach area during the study periods.

## Discussion

In the pilot study of inhaled red tide aerosol (Backer et al. 2003; Cheng et al. 2005), we showed that during the red tide episodes, nanogram per cubic meter levels of brevetoxins in the environment could be related to the observed respiratory symptoms of exposed volunteers. It was the first evidence of health effects of measured exposure to red tide aerosol. This study was designed to compare occupational exposure during a red tide aerosol episode and during a nonexposure period. PbTx-2 and PbTx-3 were the most abundant species of brevetoxins in both water and air samples, similar to what we observed in the pilot studies (Cheng et al. 2005; Pierce et al. 2003). PbTx-2 and PbTx-3 were the major species produced by *K. brevis*. With improved LC-MS analysis, we detected PbTx-1, PbTx-6, and PbTx-9 at lower concentrations. We also identified trace amounts of a brevetoxin antagonist (brevenal) produced by *K. brevis* (Bourdelais et al. 2004) that appears to inhibit the effects of brevetoxins in animal models. Identification of specific brevetoxins and the brevenal antagonist compounds in marine aerosol would help explain why some red tide blooms appear to have greater respiratory effects on humans than others.

In addition to the LC-MS technique, we used an ELISA assay to analyze the same sample. The LC-MS was more specific in identifying and quantifying individual compounds. However, it requires standards for the known brevetoxins for analysis and may miss other material that has not been identified. The ELISA technique had high sensitivity but was not specific for individual brevetoxins. It detected a class of compounds reactive to the specific antibody. It required a small amount of sample and did not need elaborated extraction for the analysis. We showed that in both air and water samples the brevetoxin concentrations from ELISA analysis were higher than those of LC-MS-MS analysis (Cheng et al. 2005). This was reasonable because for LC-MS-MS analysis we only detect and quantify PbTx-1, PbTx-2, PbTx-3, PbTx-6, and PbTx-9, whereas ELISA analysis also detected other compounds that were reactive to the assay. This indicated that there are other components of red tide aerosol that remain to be identified. Furthermore, the particle size distributions of red tide aerosol obtained from both analyses were similar, indicating that the PbTxs and related compounds were from the same marine aerosol or from marine aerosol that was produced from the same process.

The ELISA technique was critical for the analysis of the personal samples because of very low amounts of material collected on the substrates. The sensitivity of the technique allowed us to determine the amounts of brevetoxin and related compounds on personal samples. The positive results on personal samples provided information on personal exposure levels of red tide aerosol. It also provided a marker for exposure, a direct indication of red tide aerosol exposure. The exposure levels of these samples showed lower concentrations than the red tide aerosol concentrations obtained from high-volume samples. This could be attributed to a difference of sampling technique (high-volume vs. low-volume sampling), but it was more likely because the volunteers wearing the personal samples only spent part of the time in the exposure area. A record of activities may be required in future studies to further assess personal exposure.

We observed in the pilot studies that the water concentration of PbTx and onshore wind were important for red tide exposure. In this study we had the weather data near the water and air sampling location at Siesta Beach. The temperature and humidity were stable during the sampling periods. The wind speed and wind direction were very variable among the 5-day sampling periods. The wind direction and speed changed during the 8-hr sampling day. The variability of wind speed and direction resulted in changes of daily mean concentration of PbTx in aerosol samples during the 5-day study periods. Thus, the exposure periods in September 2001 showed low concentrations of PbTx levels in the air on 10 and 11 September 2001 despite high concentrations of *K. brevis* and PbTx in the water. More frequent sampling during the same exposure day may be needed to reflect the time profile of red tide aerosol concentration.

The daily red tide aerosol concentration varied substantially during the 5-day study period because of the changes in environmental conditions. Our empirical model showed the relative importance of water concentration, wind speed, and wind direction on the aerosol concentration. Basically the red tide aerosol concentration increased with water concentration, wind speed, and fraction of the time that the wind was in an onshore direction. The wind direction appeared to have the greatest weight for the aerosol concentration, confirming our previous anecdotal observations. However, this is an empirical correlation and additional data sets are needed to validate and improve the correlation.

The particle size distribution showed that the red tide aerosol consisted of coarse particles with the mean aerodynamic diameter in the range of 6–12 μm, similar to what we observed in previous studies (Cheng et al. 2005). The red tide aerosol may be produced by a breakup of bubbles from white-capped waves (Pierce et al. 1989a). Measured size distributions of marine aerosols showed a bimodal distribution with a peak in the fine-particle mode (0.1–0.2 μm) and another peak in the coarse-particle mode (2–30 μm) (Fitzgerald 1991). The coarse mode constitutes about 90–95% of the total mass but only 5–10% of the total number of particles. The coarse-particle mass in clean marine air is mainly composed of sea salt, with a strong dependence on wind speed. The red tide aerosol appears to be a component of marine coarse particles that may be associated with the sea salts.

Inhaled red tide particles can deposit efficiently in the respiratory tract. The deposit pattern is seen predominantly in the nasal and oral airways (75–84%) and 2–6% in the lower airways. Based on the environmental sampling, the estimated deposited dose rates were between 1 and 31 ng/hr in the ET region and 0.07 and 1.0 ng/hr in the lower airway for the September 2001 study.

The PbTx concentrations observed during the red tide episodes (including during previous studies) ranged from 1 to 80 ng/m^3^. The estimated total deposited dose rate is small, between 1 and 100 ng/hr (picomole range). However, this small dose was sufficient to contribute to the observed respiratory symptoms in the study subjects. From the 2000 study, we observed that a dose rate of 4–5 ng/hr in the ET region was associated with observed upper airway symptoms (throat irritation, nasal irritation, itchy skin). In the same study, the estimated dose rate in the lung was 0.15–0.2 ng/hr, which was not sufficient to cause lower respiratory symptoms. The estimated dose rate in the ET region for the occupational study (1–31 ng/hr) was associated with upper respiratory symptoms including eye irritation, nasal congestion, throat irritation, and cough (Backer et al. 2005). This was consistent with our observation in Texas (Cheng et al. 2005) and supported the observation that a lower dose rate of 1–4 ng/hr in the upper airways could cause upper respiratory symptoms. The estimated dose rate in the low respiratory airways (0.07–1 ng/hr) was not sufficient to cause lower respiratory symptoms, particularly in normal nonasthmatic subjects.

## Figures and Tables

**Table 1:** Summarized data for environmental conditions in Siesta Beach, Florida (mean ± SD).

Date	Temperature (°F)	Humidity (%)	Average wind speed (mph)	Maximum wind speed (mph)	Wind direction
7 Sept 2001	28.9 ± 0.9	69.5 ± 4.7	0.0 ± 0.0	0.1 ± 0.4	0.37 ± 0.55
8 Sept 2001	27.6 ± 0.6	75.0 ± 3.2	7.0 ± 2.8	10.9 ± 3.3	0.68 ± 0.30
9 Sept 2001	26.1 ± 0.7	81.5 ± 3.2	9.2 ± 3.5	13.3 ± 3.8	0.50 ± 0.47
10 Sept 2001	25.9 ± 0.7	86.3 ± 3.0	5.8 ± 2.2	9.3 ± 2.6	0.17 ± 0.36
11 Sept 2001	27.9 ± 1.9	NA	10.0 ± 2.0	16.7 ± 2.4	0.09 ± 0.16
3 May 2002	NA	NA	NA	NA	NA
4 May 2002	27.6 ± 0.6	NA	9.0 ± 1.8	12.2 ± 1.9	0.61 ± 0.43
5 May 2002	28.6 ± 0.8	65.8 ± 4.8	7.1 ± 3.0	10.1 ± 3.2	0.52 ± 0.42
6 May 2002	29.1 ± 2.7	56.9 ± 14.3	7.4 ± 1.9	13.2 ± 3.3	0.00 ± 0.02
7 May 2002	27.3 ± 1.2	69.8 ± 6.1	6.6 ± 0.8	9.3 ± 1.1	0.73 ± 0.42

NA, not applicable.

**Table 2:** Summarized air and water concentration of PbTxs by LC-MS method (mean ± SD).

	Siesta Beach		Lido Beach	
Date	Water concentration (μg/L)	Air concentration (ng/m^3^)	Water concentration (μg/L)	Air concentration (ng/m^3^)
7 Sept 2001	27.9 ± 14	7.53 ± 3.86	26 ± 16	26.90 ± 17.54
8 Sept 2001	18.9 ± 8	9.94 ± 6.41	18.3 ± 12.8	20.36 ± 27.16
9 Sept 2001	8.6 ± 3.7	11.89 ± 7.07	9.3 ± 6.6	17.43 ± 9.60
10 Sept 2001	10 ± 3.3	2.40 ± 2.64	13.8 ± 5	5.93 ± 7.26
11 Sept 2001	12.3 ± 2.3	1.90 ± 1.66	8.2 ± 2.4	1.32 ± 2.64
3 May 2002	0.04 ± 0.04	1.11 ± 0.48	< LOQ	0.08 ± 0.17
4 May 2002	0.3 ± 0.4	1.16 ± 0.17	< LOQ	0.08 ± 0.17
5 May 2002	< LOQ	0.05 ± 0.11	< LOQ	0.04 ± 0.09
6 May 2002	< LOQ	< LOQ	< LOQ	< LOQ
7 May 2002	< LOQ	0.06 ± 0.14	< LOQ	0.03 ± 0.06

LOQ, limit of quantitation.

**Table 3:** Summary of particle size distribution.

	Siesta Beach				Lido Beach			
	LC-MS		ELISA		LC-MS		ELISA	
	MMAD (μm)	GSD	MMAD (μm)	GSD	MMAD (μm)	GSD	MMAD (μm)	GSD
7 Sept 2001	5.97	1.73	7.02	1.81	10.85	1.89	8.33	1.65
8 Sept 2001	9.06	1.85	9.69	1.87	8.90	1.76	8.90	1.56
9 Sept 2001	10.32	1.91	11.82	1.71	8.18	1.43	8.82	1.67
10 Sept 2001	12.21	1.77	9.61	1.98	10.73	1.78	8.04	1.68
11 Sept 2001	10.20	1.73	10.87	2.05	7.59	1.95	7.45	1.83
Mean ± SD	9.55 ± 2.30	1.80 ± 0.08	9.80 ± 1.80	1.88 ± 0.13	9.25 ± 1.48	1.76 ± 0.20	8.31 ± 0.59	1.68 ± 0.10

**Table 4:** Dose rate of PbTx (nanograms per hour) in the human respiratory tract based on aerosol concentration of 1 ng/m^3^ (mean ± SD).

	Extrathoracic	TB	Pulmonary	Total
Oct 2000 (Corpus Christi, TX)	1.25 ± 0.03	0.03 ± 0.01	0.02 ± 0.01	1.30 ± 0.04
Sept 2001 (Siesta Beach, FL)	1.18 ± 0.05	0.03 ± 0.01	0.02 ± 0.01	1.23 ± 0.08
Sept 2001 (Lido Beach, FL)	1.19 ± 0.05	0.03 ± 0.01	0.02 ± 0.01	1.24 ± 0.05
